# Effects of increased precipitation on the life history of spring- and autumn-germinated plants of the cold desert annual *Erodium oxyrhynchum* (Geraniaceae)

**DOI:** 10.1093/aobpla/plz004

**Published:** 2019-02-05

**Authors:** Yanfeng Chen, Xiang Shi, Lingwei Zhang, Jerry M Baskin, Carol C Baskin, Huiliang Liu, Daoyuan Zhang

**Affiliations:** 1 Key Laboratory of Biogeography and Bioresource in Arid Land, Xinjiang Institute of Ecology and Geography, Chinese Academy of Sciences, Urümqi, China; 2 University of Chinese Academy of Sciences, Beijing, China; 3 College of Agriculture, Shihezi University, Shihezi, China; 4 College of Grassland and Environment Sciences, Xinjiang Agricultural University, Xinjiang Key Laboratory of Soil and Plant Ecological Processes, Urümqi, China; 5 Department of Biology, University of Kentucky, Lexington, KY, USA; 6 Department of Plant and Soil Sciences, University of Kentucky, Lexington, KY, USA; 7 Yili Botanical Garden, Xinjiang Institute of Ecology and Geography, Xinyuan, China; 8 Turpan Eremophytes Botanical Garden, Chinese Academy of Sciences, Turpan, China

**Keywords:** Climate change, cold desert, ephemeral (annual) plant life history, *Erodium oxyrhynchum*, increased precipitation

## Abstract

Future increased precipitation in cold desert ecosystems may impact annual/ephemeral plant species that germinate in both spring and autumn. Our primary aim was to compare the life history characteristics of plants from spring-germinating (SG) and autumn-germinating (AG) seeds of *Erodium oxyrhynchum.* Plants in field plots with simulated increases in precipitation of 0, 30 and 50 % in spring and summer were monitored to determine seedling survival, phenology, plant size, seed production and biomass accumulation and allocation. Germination characteristics were determined in the laboratory for seeds produced by plants in all increased precipitation treatments. Increased precipitation in spring significantly improved survival of seedlings from SG and AG seeds, but survival was less for AG than SG. An increase in precipitation increased the number of seeds per plant for both SG and AG, but AG produced more seeds per plant than SG. With increased precipitation, percentage of dormant seeds from SG increased significantly, while that of AG decreased slightly. Our study suggests that with increased spring and summer rainfall AG will produce an increased number of nondormant seeds that could germinate in autumn and SG an increased number of dormant seeds that become part of the soil seed bank. However, ability of some seeds to germinate in autumn and others in spring will be maintained as long as soil moisture is limited in autumn.

## Teaser

Increased precipitation prolonged the life cycle of spring and autumn germinated plants, increased dry mass accumulation and seed production, increased the proportion of biomass allocated to stems and leaves and decreased the proportion of reproduction and roots. In the context of climate change, the increase of biomass and seed production in spring and autumn germinated plants enhances the overall competitive advantage of Erodium oxyrhynchum in the plant community, assuming that other species respond less positively than Erodium oxyrhynchum.

## Introduction

Due to climate warming, the global and regional water cycle is changing, and this will have a great impact on terrestrial ecosystems (Stocker *et al.* 2013). Since plants are a critical part of terrestrial ecosystems, changed water cycles inevitably will affect their growth and reproduction (Prieto *et al.* 2008; Husen *et al.* 2017). In particular, the effects of increased/decreased amount of precipitation on the different life history stages of plants are an important issue in ecological research (Bai *et al.* 2008; Franks and Weis 2008; Gornish *et al.* 2015). Overall, a change in amount and/or pattern of precipitation can affect most life history characteristics of plants (Lu *et al.* 2014), but plant responses may vary with the species (Hayden *et al.* 2010; Ensslin and Fischer 2015; Gao *et al.* 2015).

Increased precipitation can significantly increase (Zheng *et al.* 2005; Arfin-Khan *et al*. 2018), have no effect on (Weltzin and Mcpherson 2000) or decrease (Zhao *et al.* 2012) seedling emergence (i.e. germination). During the vegetative growth stage, increased precipitation can promote the production of new leaves and branches (Zhang *et al.* 2015) and increase the number of leaves, flowers and fruits (Liu *et al.* 2012). Biomass accumulation of annual species had a significant linear increase with increased rainfall (Shem-tov and Gutterman 2003). However, the annual nondesert species *Polygonum cascadense* allocated more biomass to reproductive organs in dry than in wet years (Hickman 1975). With an increase in amount of precipitation, below-ground biomass increased in the perennial rhizomatous grass *Leymus chinensis*, while above-ground biomass increased in the perennial bunchgrass *Stipa grandis* (Liu *et al.* 2012). In addition, the maternal plant environment may cause a change in germination percentage and rate of seeds (Cendán *et al.* 2013). Seeds produced in cool wet years often are more dormant than those produced in dry warm years (Eslami *et al.* 2012; Baskin and Baskin 2014).

In arid and semi-arid regions, moisture is the most important limiting factor for plant growth, and precipitation (including snowmelt) is the main source of moisture (Sala *et al.* 1997; Ehleringer *et al.* 1998; Dube and Pickup 2001; Flannigan *et al.* 2016). In cold arid and semi-arid regions (i.e. cold deserts), plant species can be winter annuals and/or spring ephemerals, summer annuals, herbaceous perennials or woody perennials (Yuan and Tang 2010). Winter annuals and/or spring ephemerals have fast growth rates, high light use efficiency and allocate a high percentages of biomass to seeds (Yuan and Tang 2010). They complete their life cycle quickly by utilizing winter snowmelt and spring rainfall (Mao and Zhang 1994; Lu *et al.* 2014, 2016). Thus, winter annuals and/or spring ephemeral (annual) plants are very sensitive to changes in amount of precipitation.

Some annual/ephemeral species in the cold deserts of northwest China have two germination seasons: autumn and spring. When rainfall is abundant in autumn, seeds germinate in autumn and plants overwinter as rosettes and complete their (winter annual) life cycle the following spring (Lu *et al.* 2012, 2014, 2016). When precipitation in autumn is scarce, seed germination is delayed until the following spring, at which time water from snowmelt wets the soil, and the plants quickly complete their (spring ephemeral) life cycle in spring and early summer (Lu *et al.* 2012). This germination strategy may be a form of bet hedging that spreads the risk of mortality in the extreme environment of the cold deserts of northwest China (Lu *et al.* 2016). Examples of annual species whose seeds germinate in both autumn and spring in this desert include *Plantago minuta* (Zhang *et al.* 2007), *Eremopyrum distans* (Wang *et al.* 2010), *Astragalus arpilobus* (Long *et al.* 2012), *Isatis violascens* (Zhou *et al.* 2015) and *Diptychocarpus strictus* (Lu *et al.* 2016).

Spring-germinating (SG) and autumn-germinating (AG) plants of a species differ significantly in most life history characteristics, including post-germination life span (Lu *et al.* 2016, 2014), size (Zhang *et al.* 2007; Wang *et al.* 2010; Lu *et al.* 2016), fruit production (Zhang *et al.* 2007; Wang *et al.* 2010; Lu *et al.* 2016), reproductive biomass (Zhang *et al.* 2007; Lu *et al.* 2012, 2014, 2016) and seed dormancy of offspring (Wang *et al.* 2010). Plasticity in the entire life cycle of SG and AG plants likely is an adaptation to unpredictable habitats, especially the effects of precipitation (Tang *et al.* 2016), and it may be a kind of bet-hedging strategy.

Annual precipitation has a clear increasing trend in the cold desert region of northwest China, and it is predicted to increase by 30 % in the next 30 years (Shi 2003; Bai *et al.* 2008; Huang *et al.* 2017). Further, fluctuations in precipitation may reach 50 % in some extreme years (Liu *et al.* 2010). Although there have been some reports about precipitation affecting the growth of annual species in the cold deserts of northwest China (Fan *et al.* 2014; Lu *et al.* 2014), most of them focussed on species whose seeds germinate in spring. The impact of precipitation changes on the SG and AG annual plants has been studied only in *Diptychocarpus strictus* (Lu *et al.* 2014), but only the effect of watered every 3 days vs. not-watered was tested.

*Erodium oxyrhynchum* (Geraniaceae) is the most common winter annual/spring ephemeral species in the Gurbantunggut Desert (Huang *et al.* 2016; Jia *et al.* 2018). It occurs in Kazakhstan, the Caucasus, Western Asia and China, where it is mainly distributed in Xinjiang. This species is common in the Gurbantunggut Desert of Xinjiang, where its coverage can reach 20–30 % in May (Xu and Huang 1988; Wang *et al.* 2003). Based on reports by Wang *et al.* (2006) and Zhang *et al.* (2007) and on field observations by the first author from 2014 to 2016, *E. oxyrhynchum* has two germination seasons in some years: spring and autumn. Spring-germinated plants complete their life cycle (spring ephemeral) in about 2 months, before the onset of summer. Autumn-germinated plants overwinter as rosettes and complete their (winter annual) life cycle the following spring, with their life cycle being about 6 months in length (Wang *et al.* 2006). In a field site in the Gurbantunggut Desert, Zhou *et al.* (2014) found that spring (annual) ephemeral species with short life cycles were more sensitive to nitrogen deposition than annual species with long life cycles. Therefore, based on significant differences in the life cycle of spring- and autumn-germinated plants, we hypothesized that the life history traits of SG plants of *E. oxyrhynchum* are more sensitive to increased precipitation than those of AG plants. To test this hypothesis, we compared the phenology, survival, morphological traits, reproductive output and biomass accumulation and allocation of plants from spring- and autumn-germinated seeds in the field. In addition, we tested germination of seeds produced under all experimental conditions to determine whether the environment of the maternal plant influenced the proportion of dormant vs. nondormant seeds produced.

## Methods

### Seed germination of study species

In preliminary studies on freshly collected seeds of *E. oxyrhynchum*, tested at 25/10 °C, 25 % of them germinated (Ban Ying *et al*., unpubl. res.). The remaining seeds were water-impermeable and thus had physical dormancy (PY). Freshly collected seeds buried in the field germinated to 20 % between July and early November, and another 5 % germinated the following March. Since 25 % of the seeds were water permeable when buried, it is assumed that soil moisture was insufficient for all the water-permeable seeds to germinate in autumn and that the remaining 5 % of the water-permeable seeds germinated when the soil became moist in late winter.

### Study site

**Figure 1.**
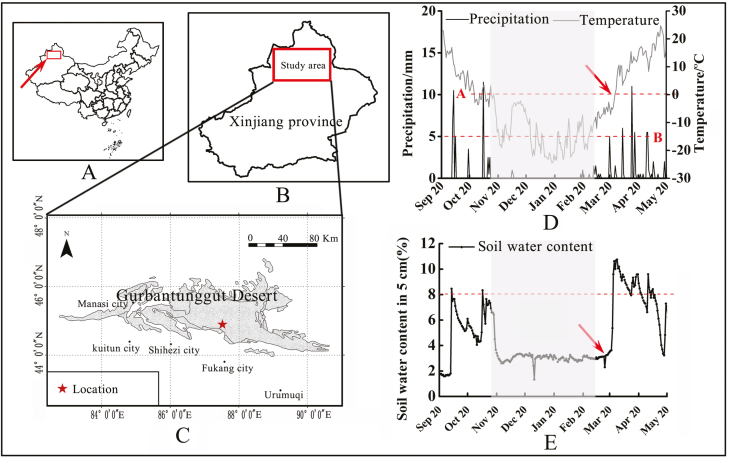
Geographical location of study site (A–C) and temperature, precipitation (D) and soil water content at 5-cm-soil depth (E) from 20 September 2016 to 20 June 2017. Shaded part of D and E indicates snowfall in winter. Red arrow (D, E) indicates when temperature reached 0 °C or higher and snow had begun to melt.

Our study was conducted on the southern margin of the Gurbantunggut Desert (44°26′N, 87°54′E) between September 2016 and June 2017 (Fig. 1A–C). The Gurbantunggut Desert (44º11′ to 46º20′N and 84º31′ to 90º00′E), covers an area of 4.88 × 10^4^ km^2^ and is the largest fixed/semi-fixed sand-dune desert in China (Wang *et al.* 2004; Yu *et al.* 2009). The extreme high and low temperatures are 42.6 and −41.6 °C, respectively; mean annual temperature is 6.6 °C. Annual precipitation is 70–180 mm, and the annual potential evaporation is about 2000 mm (Ji *et al.* 2000). In winter, there is a stable snow cover in the Gurbantunggut Desert with a maximum depth of more than 20 cm and a duration of 3–5 months (Li *et al.* 2010). The dominant plants in the Gurbantunggut Desert are the shrubs (or small trees) *Haloxylon ammodendron* and *Haloxylon persicum*, and together they cover about 30 % of the Gurbantunggut Desert, with *H. ammodendron* distributed between the dunes and *H. persicum* on top of the dunes (Huang *et al.* 2015). Herbaceous plants in this desert include *Nepeta micrantha*, *Alyssum linifolium*, *Schismus arabicus*, *Lactuca undulata*, *E. oxyrhynchum*, *Eremurus inderiensis*, *Orostachys spinosus*, *Euphorbia turczaninowii* and *Agriophyllum squarrosum* (Wang *et al.* 2004).

### Experimental procedure

**Figure 2.**
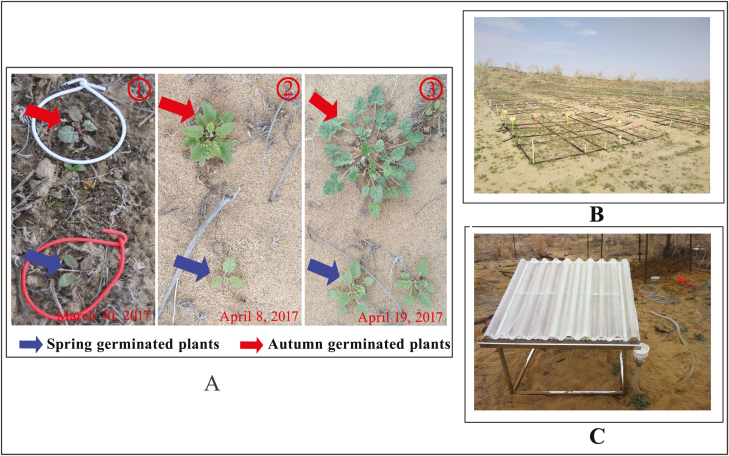
Spring- and autumn-germinating plants of *Erodium oxyrhynchum* (A), study site (B) and station to collect rainfall (C) in the field.

To simulate current, future and extreme amounts of precipitation in the Gurbantunggut Desert, we set up an experiment for 0, +30 and +50 % increase in precipitation, based on actual precipitation in spring and summer 2017 at the study site. Precipitation treatments were given to plants from SG and AG seeds. The treatments were 0 % increase in precipitation (SG_0_, AG_0_), +30 % increase in precipitation (SG_30_, AG_30_) and +50 % increase in precipitation (SG_50_, AG_50_). Each treatment was randomly assigned to one 1 × 1 m subplot within each of the eight 3 × 5 m plots; thus, there were four replicates for phenology monitoring and four for biomass sampling. The plots were distributed in a relatively homogeneous, flat inter-dune lowland (Fig. 2B). In addition, to prevent competition from other species, the plots were hand-weeded in November 2016 and after snowmelt in early April 2017. All plots were surrounded by black plastic film that extended to a depth of 80 cm into the soil and 10 cm above ground level to avoid runoff of irrigation water into non-watered areas (Fig. 2B).

We set up four stations to collect rainfall and snow in spring and summer to use in the water-addition treatments. Each station (Fig. 2C) consisted of a 4 × 4 m plastic board set at an angle of 30 ° to the ground surface. The four corners of the board were fixed to an iron pole, and a bucket was placed at the lower corner of the board to collect precipitation.

A weather and soil moisture monitoring device (Caipos GmbH, Schillerstrasse Gleisdorf, Austria) was installed at the study site on 1 September 2014 and used to monitor precipitation, temperature and soil moisture. The device collected data via wireless sensors and uploaded them to a computer every hour. According to the daily precipitation data from the monitoring device, we added water to plots the second day after a precipitation event (including some snow) to obtain +30 and +50 %. Water was collected as described above and applied with a watering can. The total precipitation during the life history cycle was 48 mm based on the monitoring device, and 12.5 and 24.0 mm water were added during the experiment for +30 and +50 %, respectively.

The density of *E. oxyrhynchum* plants in the field can reach 96.5 plants/m^2^ (field observations by the first author from 2014 to 2016), and thus the number of plants meet the needs for our experiments. We marked plants by placing a red or white wire around the stem of each SG and AG, respectively (Fig. 2A). On 10 October 2016, 12 plants were marked in each of the 1 × 1 m subplots for treatments of AG, and all other plants were removed, and on 10 March 2017, 12 plants were marked in each of the subplots for treatments of SG and other plants removed. We designated the seeds from which autumn- and SG plants were produced as the F1 generation and the seeds produced by autumn- and SG plants as the F2 generation.

### Measurements and sampling

#### Phenology

We followed the methods of Lu *et al.* (2014) on phenology. At 3-day intervals from germination to death, the plots were checked, at which time data were recorded for emergence date (i.e. number of days until all seeds in each treatment had emerged), flowering date (number of days until all plants in each treatment had flowered), fruiting date (number of days until occurrence of the first green fruit on all plants in each treatment), maturation date (number of days until the first fruit of all individuals in each treatment had turned a brown-colour and were ready to disperse naturally) and post-germination life span (emergence of the first individual until death of the last individual).

#### Survivorship

For each treatment, we recorded the number of living plants at weekly intervals, except from November 2016 to March 2017, when snow covered the plots.

#### Morphological characters

When plants reached maturity, plant height, leaf area, length of main root, number and length of branches, number of flowers and number of fruits and number of seeds were measured/determined. For leaf area, all leaves were collected and their area measured with a LI-COR 3000 leaf area meter. All branches on the main stem were counted, and length of the longest branch was measured.

#### Dry mass accumulation and allocation

After the morphological traits had been measured, plants in all treatments were harvested and separated into root, stem, leaves and reproductive organs (flowers, fruits and seeds), and the roots were carefully washed free of soil. Seeds were harvested from plants at the time of maturity, i.e. when fruits were dry, yellow and dehiscing. As a result of the different precipitation treatments, seeds matured at different times. Thus, we collected the above- and below-ground parts of each plant as soon as its seeds had matured. The fruits (without seeds), leaves, stems and roots (washed free of soil) of each plant were weighed separately after drying at 75 °C for 48 h using a Sartorius BS210S electronic-balance (0.0001g). Total biomass was calculated as the sum of roots, stems, leaves and reproductive organ (flowers, fruits and seeds) per plant. Allocation of biomass to roots, stems, leaves and each reproductive organ (flowers, fruits and seeds) was expressed as a percentage of the total dry mass.

#### Offspring seed germination

Mature fruits from different treatments of AG and SG were collected on 21 May and 1 June 2017, respectively, and the seeds removed from them. Germination tests were conducted at 25/10 °C (12/12 h), starting on 20 June 2017. Twenty-five seeds were placed in each of four 7-cm-diameter Petri dishes on two layers of Whatman No. 1 filter paper moistened with 3 mL of distilled water. Water was added as needed to keep the filter paper moist during the test period. Germinated seeds (emerged radicle) were counted and removed every day for 30 days. Final percentage of germination (FPG) was estimated as FPG = GN / SN, where GN is total number of germinated seeds and SN number of viable seeds. After the germination trials were complete, non-germinated seeds were tested for viability using TTC (2, 3, 5-Triphenyltetrazolium chloride). Seeds were cut open and placed in a 0.1 % aqueous TTC solution at 20 °C for 24 h in darkness. Embryos that stained red or pink were considered to be viable and those that did not stain non-viable (Baskin and Baskin 2014).

### Statistical analysis

Data were arcsine (percentage data) or log_10_ (other data) transformed before analysis to approximate normal distribution and homogeneity of variance. If the variance of transformed data was still not homogenous, treatment differences were assessed using the Kruskal–Wallisnon-parametric test. Tukey’s test was performed for multiple comparisons to determine significant differences among treatments, and a Bonferroni correction was performed to avoid type I error problems. Survival percentage, morphological characters (plant height, leaf area, root length, branch length and number of branches, flowers, fruits and seeds), dry mass accumulation and allocation (total dry mass and proportion allocated to roots, stems, leaves and reproductive organs) and offspring seed germination were analysed as dependent variables with a two-way ANOVA. Germination season and watering regime were considered as fixed effects. All data analyses were performed with the software SPSS 13.0 (SPSS Inc., Chicago, IL, USA). All figures were drawn with Origin software 2015 (Origin Lab, Northampton, MA, USA).

## Results

### Precipitation and temperature

From October 2016, daily average temperature decreased gradually, reached a minimum of −24.48 °C on 17 January 2017 and then rose rapidly to 26.6 °C on 25 May 2017 (Fig. 1D). Snow depth was 20.23 ± 0.40 cm at the experimental site on 16 February 2017, and snow-melting began on 20 March 2017. With the beginning of snowmelt, soil moisture increased sharply and remained above 8 % from late March to late April (Fig. 1E).

### Phenology

In the control (0 % increase in precipitation), dates of emergence, leafing, flowering, fruiting and the withered yellow stage of AG_0_ occurred significantly earlier than for SG_0_, and the life cycle of AG_0_ was significantly longer than that of SG_0_ (*P* < 0.05, Table 1). Increase in precipitation delayed flowering, fruiting and withering of AG and SG plants about 1–2 days, but the difference was not significant (Table 1).

**Table 1.** Effects of increased precipitation on life cycle phenology of spring-germinating (SG) and autumn-germinating (AG) plants of *Erodium oxyrhynchum*. SG_0_, 0 % increase in precipitation for SG; SG_30_, 30 % increase in precipitation for SG; SG_50_, 50 % increase in precipitation for SG; AG_0_, 0 % increase in precipitation for AG; AG_30_, 30 % increase in precipitation for AG; AG_50_, 50 % increase in precipitation for AG. Different lowercase letters indicate significant differences (*P* < 0.05) among 0, + 30 and + 50 % increase in precipitation for SG and AG and different uppercase letters significant differences between SG and AG at the same increase in precipitation.

	Spring-germinating plants			Autumn-germinating plants		
	SG_0_	SG_30_	SG_50_	AG_0_	AG_30_	AG_50_
Emergence	30 March	30 March	30 March	10 October	10 October	10 October
Leafing date	3 May	5 May	3 May	20 April	23 April	21 April
Flowering date	12 May	13 May	13 May	9 May	10 May	10 May
Fruiting date	14 May	15 May	14 May	11 May	13 May	12 May
Yellowing date	25 May	26 May	25 May	21 May	23 May	23 May
Life cycle (day)	66.10 ± 1.57^Ba^	66.30 ± 1.82^Ba^	67.10 ± 1.32^Ba^	234.30 ± 1.49^Aa^	235.80 ± 1.03^Aa^	236.50 ± 1.71^Aa^

### Survival

**Figure 3.**
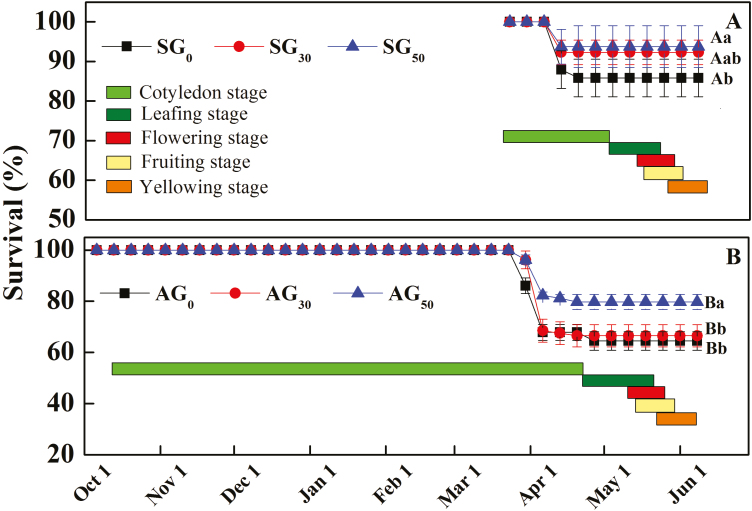
Effects of increased precipitation on survival percentage (mean ± 1 SE) of spring-germinating (SG) plants and autumn-germinating (AG) plants of *Erodium oxyrhynchum*. The treatments were 0 % increase in precipitation, including water from snowmelt (SG_0_, AG_0_), +30 % increase in precipitation (SG_30_, AG_30_) and +50 % increase in precipitation (SG_50_, AG_50_). Different lowercase letters indicate significant differences (*P* < 0.05) among 0 , + 30 and + 50 % increase in precipitation for SG and AG and different uppercase letters significant differences between SG and AG at the same increase in precipitation.

In the control, survival of AG and SG decreased sharply in late March to early April 2017 (Fig. 3A, B), and final survival of AG (63.6 %) was significantly lower than that of SG (87.9 %) (*P* < 0.05, Table 2). Increased precipitation improved survival of SG plants significantly from 87.9 (SG_0_) to 92.3 (SG_30_) and 93.8 % (SG_50_) (*P* < 0.05, Table 2). A 50 % increase in precipitation also improved survival of AG significantly (*P* < 0.05, Table 2) from 63.6 (AG_0_) to 79.7 % (AG_50_), but a 30 % increase in precipitation did not improve the survival of AG significantly (Fig. 3A, B).

**Table 2.** Summary of a two-way ANOVA showing the effects of different germination seasons and increased precipitation treatments on life history traits. P, increase in precipitation; S, germination season; P*S, increased in precipitation × germination season.

Plant traits	Source of variation	df	*F*-value	*P*-value	Plant traits	*F*-value	*P*-value
Survival	P	2	2.14	< 0.05	Stem biomass	29.502	< 0.05
	S	1	15.916	< 0.05		57.941	< 0.05
	P*S	2	0.07	0.932		1.07	0.355
Root length	P	2	3.136	0.058	Leaf biomass	11.986	< 0.05
	S	1	1.226	< 0.05		12.796	< 0.05
	P*S	2	0.853	0.436		0.86	0.433
Height	P	2	2.592	0.091	Reproduction biomass	2.627	0.088
	S	1	20.747	< 0.05		14.483	< 0.05
	P*S	2	2.537	0.095		0.955	0.396
Leaf number	P	2	1.421	0.257	Total biomass	16.489	< 0.05
	S	1	11.926	< 0.05		38.246	< 0.05
	P*S	2	3.959	< 0.05		0.931	0.405
Leaf area	P	2	10.104	< 0.05	Allocation of biomass to root	2.38	0.109
	S	1	20.672	< 0.05		8.753	< 0.05
	P*S	2	2.402	0.107		0.866	0.431
Branch number	P	2	0.988	0.384	Allocation of biomass to stem	4.988	< 0.05
	S	1	6.999	< 0.05		5.396	< 0.05
	P*S	2	2.533	0.096		0.459	0.636
Branch length	P	2	1.111	0.342	Allocation of biomass to leaf	4.051	< 0.05
	S	1	7.251	< 0.05		9.74	< 0.05
	P*S	2	3.877	< 0.05		2.297	0.117
Seed number	P	2	11.161	< 0.05	Allocation of biomass to reproduction	3.084	0.06
	S	1	14.78	< 0.05		1.061	0.311
	P*S	2	0.981	0.386		0.571	0.571
Root biomass	P	2	5.565	< 0.05	Germination percentage	7.113	< 0.05
	S	1	6.49	< 0.05		174.194	< 0.05
	P*S	2	0.475	0.626		23.661	< 0.05

### Morphological characters

**Figure 4.**
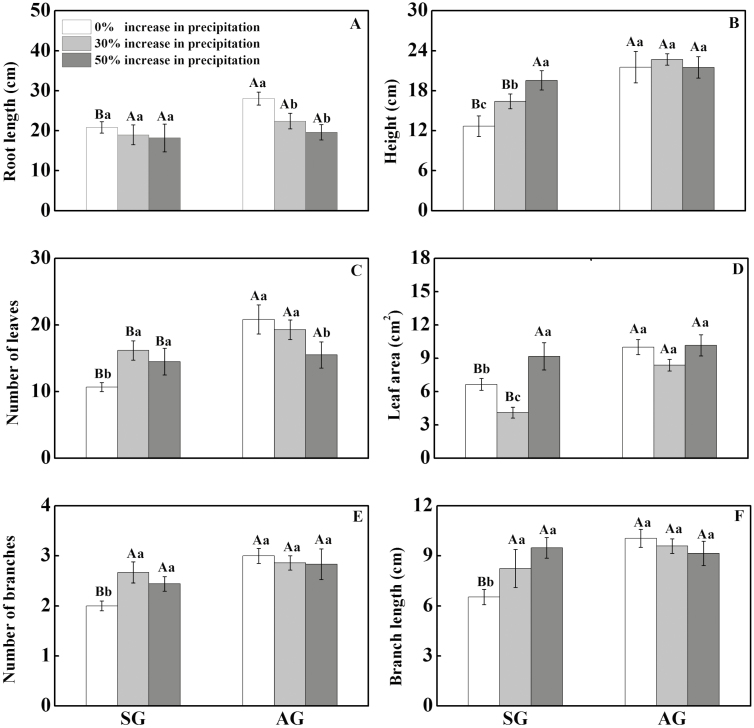
Effects of increased precipitation on root length (A), height (B), leaf number (C), leaf area (D), branch number (E) and branch length (F) of spring-germinating (SG) plants and autumn-germinating (AG) plants of *Erodium oxyrhynchum*. Different lowercase letters indicate significant differences (*P* < 0.05) among 0, + 30 and + 50 % increase in precipitation for SG and AG and different uppercase letters significant differences between SG and AG at the same increase in precipitation.

In the control, root length, plant height, leaf number, leaf area and number and length of branches differed significantly between SG and AG (*P* < 0.05, Table 2), i.e. AG > SG (Fig. 4A–F). Increased precipitation significantly increased plant height, number and area of leaves and number and length of branches of SG (*P* < 0.05, Table 2). However, there was no significant change in root length of SG with increased precipitation. For AG, increased precipitation (+30 and +50 %) decreased root length and leaf number significantly (*P* < 0.05, Table 2), but it had no effect on plant height, leaf area or number and length of branches (Fig. 4A–F).

### Seed production

**Figure 5.**
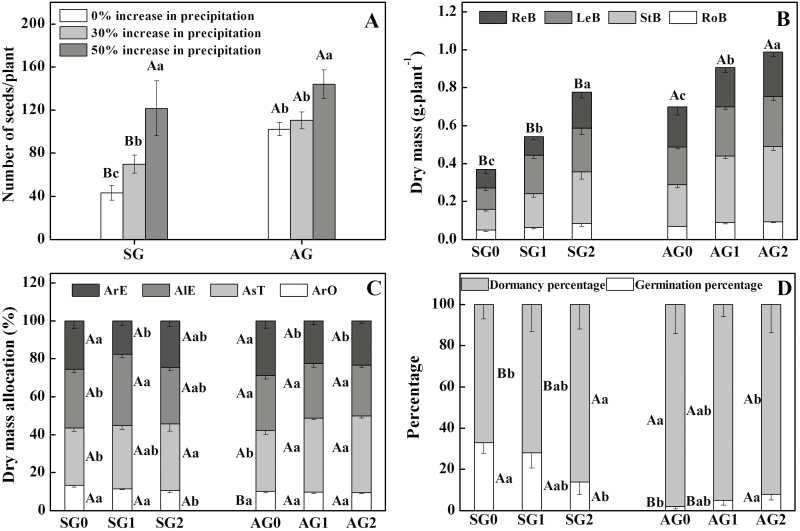
Effects of increased precipitation on seed number (A), dry mass (B), dry mass allocation (C) and offspring seed germination (D) of spring-germinating (SG) and autumn-germinating (AG) plants of *Erodium oxyrhynchum*. Different lowercase letters indicate significant differences (*P* < 0.05) among 0, + 30 and + 50 % increase in precipitation for SG and AG and different uppercase letters significant differences between SG and AG at the same increase in precipitation. ReB, reproductive biomass; LeB, leaf biomass; StB, stem biomass; RoB, root biomass; ArO, allocation of biomass to roots; AsT, allocation of biomass to stems; AlE, allocation of biomass to leaves; ArE, allocation of biomass to reproductive organ.

Number of seeds produced by AG_0_ was significantly higher than that produced by SG_0_ (*P* < 0.05, Table 2 and Fig. 5A). Increased precipitation (+30 and +50 %) significantly increased the number of seeds produced by AG and SG (*P* < 0.05, Table 2). Seed number per SG plant increased from 45 (SG_0_) to 72 (SG_30_) and 122 (SG_50_), and seed number per AG plant increased from 102 (AG_0_) to 110 (AG_30_) and 142 (AG_50_) (Fig. 5). With increased precipitation (+30 and +50 %), number of seeds produced by AG was also significantly higher than that produced by SG (*P* < 0.05, Table 2 and Fig. 5A).

### Dry mass accumulation and allocation

Total dry mass of AG_0_ was 1.7 times that of SG_0_, i.e. 0.368 and 0.629 g per plant, respectively. Similarly, dry mass of reproductive organs of AG_0_ was 1.8 times than that of SG_0_ plants, i.e. 0.097 and 0.176 g per plant, respectively (Fig. 5B). Increased precipitation had a significant effect on accumulation of total biomass of SG and AG (*P* < 0.05, Table 2). Total biomass per plant for SG was 0.777 (SG_30_) and 0.894 g (SG_50_), and for AG it was 0.813 (AG_30_) and 0.943 g (AG_50_) (Fig. 5B). Total biomass of AG differed significantly from SG at 0 and 30 % increased precipitation (*P* < 0.05, Table 2), but the difference was not significant at 50 % increased precipitation. Reproductive allocation did not differ significantly between SG and AG. With increased precipitation, reproductive and root allocation generally decreased in SG and AG (Fig. 5C), but the difference was not significant. There was a trend for increasing allocation to stems and leaves in SG and AG, and for SG it was significant (Fig. 5C).

### Offspring seed germination

Germination of F_2_ seeds produced by SG_0_ and AG_0_ was 33.0 and 2.0 %, respectively (*P* < 0.05, Table 2 and Fig. 5D). With increased precipitation, germination percentage of seeds produced by SG decreased significantly (*P* < 0.05, Table 2) to 28.0 (SG_30_) and 14.0 % (SG_50_), whereas germination of seeds produced by AG increased only slightly to 5.0 (AG_30_) and 8.0 % (AG_50_) (Fig. 5D).

## Discussion

Our hypothesis that the life history traits of SG plants of *E. oxyrhynchum* are more sensitive than those of AG plants in response to increased precipitation in the field in spring was supported by some but not all results. Increased precipitation increased seed production and total biomass more for SG than for AG plants, thus supporting our hypothesis. Root length of SG was not increased or decreased by a 30 or 50 % increase in precipitation, whereas that of AG was decreased significantly (Fig. 3A, B). Thus, root length data do not support our hypothesis.

The overall percentage of survival to maturity of AG was lower than that of SG in the control, probably due to AG experiencing extremely low temperatures in winter (Fig. 1D), which caused severe damage leading to plant death. This finding is consistent with the results for seedlings of *Chamaecyparis nootkatnesis* in which the exposure to freezing stress damaged the fine roots and foliar browning and mortality occurred after the onset of warming conditions (Schaberg *et al.* 2010). Also, some SG and AG plants died in early April (Fig. 3A, B). Plant death in April likely can be attributed to frost-damaged tissues not becoming metabolically active as temperatures increased in spring (Fan *et al.* 2014). Also, there were some late freezes in April (Fig. 1D), if plants had lost their cold hardiness a late freeze may have killed them (Knight and Heather 2012). Further, the different effects of increased precipitation on survival of SG and AG are related to differences in phenology stage and size of SG and AG when precipitation was increased in spring. SG were still in the seedling stage with only a few leaves, while AG were larger with more leaves and thus not as sensitive as SG to increased precipitation. Therefore, future increased precipitation in early spring may especially improve the survival of SG.

The morphological characteristics of AG and SG in response to increased precipitation only partly support our hypothesis. That is, increased precipitation significantly increased all of the measured morphological characters of SG, except root length, while it increased some characters of AG and decreased others. The significant decrease in root length of AG_30_ and AG_50_ compared with AG_0_ indicates that increased water in the upper soil profile stimulated roots to stop growing into the deeper layers. However, the relatively dry soil of AG_0_ stimulated roots to grow deeper into the soil. Studies on *Stipa bungeana* in the desertified grasslands of the Ordos Plateau in Inner Mongolia (China) also have found that root length increased as depth to moist soil in the soil profile increased (Cheng *et al.* 2006). Longer roots of AG than SG means that AG could absorb water from a greater soil depth than SG, and AG is less dependent on surface water than SG. Thus, AG may have an advantage over SG in relatively dry springs.

When precipitation was increased, seed production of SG was significantly higher than that of AG, which supports our hypothesis that the response of SG is more sensitive to increased precipitation than that of AG. In *Eremopyrum distans* (Wang *et al.* 2010) and *Diptychocarpus strictus* (Lu *et al.* 2014), plants derived from autumn-germinated seeds produced more seeds than those from spring-germinated seeds. The reason for the higher seed production in AG than in SG is that AG accumulated more resources for reproduction than SG. Thus, the difference in seed production of AG and SG of *E. oxyrhynchum* leads to different contributions to the soil seed bank. In the context of increased precipitation, the capability of SG and AG to produce more seeds that potentially remain viable in the soil allows them to form large persistent seed banks, thereby spreading germination risk over time. This is similar to the increased seed production in wet vs. dry years of plant species growing in unpredictable desert environments (Marone *et al.* 2000; Quevedo-Robledo *et al.* 2010).

With an increase in precipitation, both SG and AG accumulated more biomass than the controls, which does not support our hypothesis. Increased precipitation promoted growth of stems and production of leaves, fruits, seeds and total biomass of both AG and SG, as has been shown in some hot (Shem-tov and Gutterman 2003) and cold (Yu *et al.* 2009) desert species that plants were larger and produced more seeds under irrigation regimes). However, the increase in biomass of SG was significantly greater than that of AG, which supports our hypothesis. This difference between AG and SG may have been caused by a combination of factors. First, the phenological stage strongly influences the responses of plants to the environment, and generally the earlier the phenological stages the more sensitive plants are to environmental changes (Petraglia *et al.* 2014). Second, since AG were significantly larger than SG when water was added, the addition of water to AG may have been insufficient to elicit a strong response in growth and biomass accumulation. This difference in biomass accumulation of SG and AG is also consistent with the response of the morphological traits (height, leaf number and area and branch number and length) of SG and AG to increased precipitation. Therefore, SG showed a more obvious growth plasticity response to increased precipitation than AG.

With increased precipitation, the proportion of biomass allocated to stems and leaves of SG and AG increased, and the proportion allocated to reproduction and roots decreased. According to the theory of optimal allocation, plants allocate more resources to aboveground than to belowground organs, which allows them to obtain additional space and light and consequently to increase their competitiveness and productivity (Mccarthy and Enquist 2007). However, SG were more efficient than AG in increasing reproductive fitness. Since the life cycle of AG is longer than that of SG and AG are larger than those of SG, it is expected that seed production would be greater in AG than in SG. However, increased precipitation did not have much effect on seed yield of AG via adjustment of reproductive allocation, while it promoted growth (but not seed production) of SG. Moreover, other environmental conditions (especially, increased temperature) in nature may accelerate entrance of SG into the reproductive stage before they reached a large size (Espe *et al.* 2017). As a result, the proportion of stems and leaves increased, and the proportion of reproduction decreased in SG.

AG produced proportionally more seeds with PY than SG, while SG produced proportionally more nondormant (ND) seeds than AG (Fig. 5D). The significant differences of SG and AG in seed dormancy may provide two completely different directions for species evolution. Nondormant seeds such as those of *E. oxyrhynchum* can germinate rapidly and occupy space when water first becomes sufficient to promote germination. However, if drought follows seedlings may die. Dormancy ensures that there is a reserve of seeds that can germinate at some time in the future when seedlings potentially will be able to survive (Baskin and Baskin 2014). However, if the soil is dry in autumn, germination of the ND seeds of *E. oxyrhynchum* will be delayed until snowmelt in spring, giving rise to SG. Thus, depending on how late in the growing season rain occurs, there could be an increase in AG or SG. With an increase in AG and SG, and thus an increase in seeds with PY and ND, respectively, and number of seeds in the seed bank and the number of plants in the standing vegetation will increase, respectively. Thus, in the context of increased precipitation, the differences of SG and AG in seed dormancy not only avoid the risk of population extinction from a one-time germination event, but also enhance competitivenessof the species in the plant community.

## Conclusions

For both AG and SG, increased precipitation prolonged the life cycle; increased dry mass accumulation and seed production; increased the proportion of biomass allocated to stems and leaves; and decreased the proportion of reproduction and roots. These results do not support our hypothesis that the response of SG is more sensitive to additional water than AG. In the context of climate change, the increase of biomass and seed production in SG and AG enhances the overall competitive advantage of *E. oxyrhynchum* in the plant community, assuming that other species respond less positively than *E. oxyrhynchum*. The results on survival and morphological characteristics of AG and SG partly support our hypothesis. Thus, with increasing precipitation, SG accumulated proportionally more biomass and produced proportionally more seeds per plant than AG. AG produced proportionally more dormant seeds than SG, while SG produced proportionally more ND seeds than AG, which supports our hypothesis. The effect of increased precipitation with climate change could modify the proportion of dormant and nondormant seeds produced by SG and AG. Since SG and AG produced both dormant and nondormant seeds when precipitation was increased, it seems likely that *E. oxyrhychum* will continue to produce autumn- and spring cohorts of plants even with increased summer precipitation. In addition to increased precipitation, the effects of other factors such as increased temperatures, which would advance reproductive phenology, and nitrogen deposition, which would promote plant growth, of desert plants cannot be ignored (Brooks 2003; Wertin *et al.* 2015; Huang *et al.* 2018). Both increased temperatures and nitrogen deposition have the potential to influence the ND:PY ratio of seeds produced by SG and AG (Baskin and Baskin 2014).

## Sources of Funding

Funds for this study were provided by the National Natural Science Foundation of China (31570529, 31660162, 31770638, U1503101) and Youth Innovation Promotion Association of Chinese Academy of Sciences (2018477).

## Contributions by the Authors

Y.C., H.L. and D.Z. conceived and designed the study; Y.C., H.L. and L.Z. performed most of the experiments; Y.C., H.L. and X.S. analysed the data. Y.C., H.L., X.S., D.Z., J. B, C. B. and L.Z. wrote the manuscript.

## Conflict of Interest

None declared.

## Supporting Information

The original data information of figures and tables is available online (https://osf.io/3vndh/).
